# Notes and News

**Published:** 1902-04-26

**Authors:** 


### Notes and News.

The foundation-stone of a cottage hospital at Maryport, Cumberland, has been laid.

All the societies for the prevention of tuberculosis in France have been federated under the presidency of Professor Brouardel.

A temporary small-pox hospital has been erected at Elswick. It has been built from plans prepared by Dr. Sergeant, and will be used eventually to supplement a permanent structure in course of erection. The temporary hospital cost £600; the permanent one is estimated at £18,000.

The Rev. Canon Fleming, B.D., and Mr. Frank T. Bullen, have promised to speak at Exeter Hall on Tuesday afternoon, 29th inst., on the occasion of the fifty-ninth annual meeting of the National Refuges and Training Ships *Arethusa* and *Chichester*, when the Earl of Jersey, President, will take the chair, and a choir of children and sailor boys will sing. Tickets may be obtained of the secretary, Mr. H. Bristow Wallen, at 164 Shaftesbury Avenue, W.C.

An interesting paper was recently read by Dr. Doyen before the Académie de Médecine giving an account of the operation which he undertook for the separation of the xiphopagous twins, Radica and Doodica. It appeared that notwithstanding the individuality of the two "twins," so far as many of their characteristics were concerned, their circulation was in common, a fact which could be easily demonstrated, for by the use of methyline blue it could be shown that an interchange of blood took place very quickly. An important point in relation to the arrangement of anastomosing arteries in the bond of union between them was that Radica obtained more arterial blood than her sister, and to this was attributed her greater strength, for Doodica was always the weaker of the two. On the other hand, the sudden deprivation of this portion of her blood supply probably accounted for the feebleness shown by Radica after the operation, while Doodica appeared for a time to become more resistant. The death of Doodica was due to the tuberculous peritonitis with which she was affected. But for the lateral curvature caused by the prolonged constraint in an abnormal position Radica is now quite well.

A new workhouse infirmary at Shirley Warren has been opened by the Southampton Board of Guardians. The infirmary consists of three pavilions for men, two for women, a maternity and an isolation block, besides the usual administrative buildings, offices, and nurses' home. The accommodation is for 312 patients. The cost amounted to about £87,000.

Inquiries have been made in the House of Commons as to the results of anti-typhoid innoculation amongst the troops in South Africa. The reply was that a report dealing with less than 5,000 cases had been received, which, although it gave evidence of a lower mortality amongst the innoculated, was not regarded as sufficiently conclusive to be the subject of any statement.

In a letter to the chairman of the Royal Infirmary, Aberdeen, Lord Mount-Stephen makes the munificent offer to assist in supplying the annual need of the infirmary for a further £3,000 per annum by contributing £1,000 per annum, on the understanding that the working people and other inhabitants of Aberdeen shall also make special efforts to provide the £2,000 still required.

The director of the Paris Assistance Publique has made arrangements whereby treatment will be extended to patients in their own homes in connection with the hospitals when the case is not urgent, but at the same time suitable for hospital treatment. The arrangement is intended to relieve the overcrowding in the hospitals, which at the present time causes cases of urgency to be refused admission. The new system will permit of selection being made, and the earlier return of many cases to the home.

The bazaar in aid of the Hospital for Sick Children to be held at Coronation time will take place in the Botanical Gardens, instead of in the garden of the hospital in Great Ormond Street. It is not to be wondered at that the more extensive area has been selected, as the bazaar has assumed very large proportions. The list of stallholders is very distinguished, and the stalls will number twenty-five. Russia, America, Germany, South Africa, and Australia each have separate sections, whilst the Stock Exchange stall will be one of the greatest novelties.

Professor Whitla, President of the Ulster Medical Society, has earned the gratitude of all medical men in Ulster and the North of Ireland by his munificence and generosity in providing and equipping a medical institute in Belfast, the foundation stone of which was recently laid by Dr. G. Redfern in the presence of a large number of members of the medical profession. The cost will be about £5,000. The building is situated in College Square North, and will be fitted up in the most complete manner, so as to render it a fit meeting place for the Ulster Medical Society, whose property it will ultimately become. Indeed, when we hear of it containing not only library and lecture-hall, but billiard-room, kitchen, drawing-rooms, etc., we cannot but compare the professional sociability which these things would appear to connote with the severe austerity of scientific meetings in London.

